# Hepatitis B virus infection and its associated factors among medical waste collectors at public health facilities in eastern Ethiopia: a facility-based cross-sectional study

**DOI:** 10.1186/s12879-021-05918-x

**Published:** 2021-02-27

**Authors:** Degu Abate Mengiste, Abebe Tolera Dirbsa, Behailu Hawulte Ayele, Tewodros Tesfa Hailegiyorgis

**Affiliations:** 1 Department of Medical Laboratory Sciences, College of Health and Medical Sciences, Haramaya University, P.O. Box, 235, Harar, Ethiopia; 2 Epidemiology and Biostatistics Unit, College of Health and Medical Sciences, School of Public Health, Haramaya University, P.O. Box, 235, Harar, Ethiopia; 3 Public Health and Policy Unit, College of Health and Medical Sciences, School of Public Health, Haramaya University, P.O. Box, 235, Harar, Ethiopia; 4 Department of Medical Laboratory Sciences, College of Health and Medical Sciences, Haramaya University, P.O. Box, 235, Harar, Ethiopia

**Keywords:** Seroprevalence, Occupational exposure, Medical waste, Sharps injuries, Eastern Ethiopia

## Abstract

**Background:**

The risk of hepatitis B virus infection among medical waste handlers who undergo collection, transportation, and disposal of medical wastes in the health institutions is higher due to frequent exposure to contaminated blood and other body fluids. There is limited evidence on the seroprevalence of hepatitis B among medical waste handlers in eastern Ethiopia. The study was aimed at studying the seroprevalence of Hepatitis B Virus and associated risk factors among medical waste collectors at health facilities of eastern Ethiopia.

**Methods:**

A facility-based cross-sectional study was conducted among randomly selected medical waste collectors from public health facilities in eastern Ethiopia from March to June 2018. A pre-tested and well-structured questionnaire was used to collect data on socio-demographic characteristics and hepatitis B infection risk factors. A2.5ml venous blood was also collected, centrifuged and the serum was analyzed for hepatitis B surface antigen using the instant hepatitis B surface antigen kit. Descriptive summary measures were done. Chi-square and Fisher exact tests were used to assess the risk of association. Multivariate logistic regression was conducted with 95% CI and all value at *P*-value < 0.05 was declared statistically significant.

**Results:**

From a total of 260 (97.38%) medical waste collectors participated, HBV was detected in 53 (20.4%) of the participants [95%CI; 15.8, 25.6]. No significant differences were observed in the detection rates of HBV with respect to socio-demographic characteristics. In both bivariate and multivariable logistic regression analysis, being unvaccinated (AOR = 6.35; 95%CI = [2.53–15.96], *P* = 0.001), history of blood transfusion (receiving) (AOR; 3.54; 95%CI; [1.02–12.24], *P =* 0.046), history of tattooing (AOR = 2.86; 95%CI = [1.12–7.27], *p* = 0.03), and history of multiple sexual partner (AOR = 10.28; 95%CI = [4.16–25.38], P = 0.001) remained statistically significantly associated with HBsAg positivity.

**Conclusion:**

This cross-sectional study identified that HBV infection is high among medical waste collectors in eastern Ethiopia. Immunization and on job health promotion and disease prevention measures should be considered in order to control the risk of HBV infection among medical waste collectors in eastern Ethiopia.

**Supplementary Information:**

The online version contains supplementary material available at 10.1186/s12879-021-05918-x.

## Introduction

Improper management of waste produced in the course of healthcare activity carries a high risk of environmental hazards and public health risks [1–3]. Occupational exposure to percutaneous needle sticks injuries during segregation or recapping, for example, pose a significant risk of occupational transmission of blood borne pathogens such as human immunodeficiency virus (HIV), hepatitis B virus (HBV) and hepatitis C virus (HCV) particularly among medical waste handlers (MWHs) [4–6]. Hepatitis B virus causes liver inflammation, which may lead to fibrosis, cirrhosis, or liver cancer [7, 8] and it is usually transmitted through contact with infected body fluids, such as blood, semen, and percutaneous injuries [9].

Estimates from the World Health Organization (WHO) indicate that in 2015, an estimated 257 million people were living with chronic HBV infection. Chronic HBV epidemics mostly affected the WHO African Region and the Western Pacific Region which account for 68% of those infected. Furthermore, mortality from viral hepatitis has increased by 22% since 2000 [10]. Some literature in Ethiopia indicates that HBV infection among medical waste collectors ranges between 6 and 6.3% [11, 12] and an overall pooled prevalence of 8% HBV among the community [13]. Hepatitis B vaccine coverage with the initial birth dose vaccination is still low at 39% and other prevention interventions are insufficiently implemented [10].

Medical waste handlers are highly involved in health care waste management including waste segregation, transportation, storage, treatment, and final disposal of all types of waste generated in the health care facilities that require a high standard of training [14, 15]. High prevalence of HBV infection occurred in public health center cleaners (PHCCs) and exposure increased with the amount of waste generated that increase as the number of patients flow increases [16].

This was reported in some cases mainly due to a high level of non-compliance to standard medical waste management procedures [17] or unavailability of post-exposure prophylaxis [18, 19]. Despite national and international efforts to minimize occupational hazard through raising awareness and seeking solutions to the problems associated with healthcare waste management for many years, health care waste management (HCWM) have failed and health care workers particular medical waste handlers are continuing to be at increased risk of HBV infection [15, 20]. Reports indicate that access to affordable hepatitis testing is limited and only a few people with viral hepatitis have been diagnosed (9% of HBV infected persons) [10]. Information on the spread of infection resulting from waste handling in eastern Ethiopia is limited. Therefore, this study was conducted to identify the magnitude of HBV infection among MWHs and its associated factors.

## Methods and materials

### Study design and setting

**Fig. 1:**
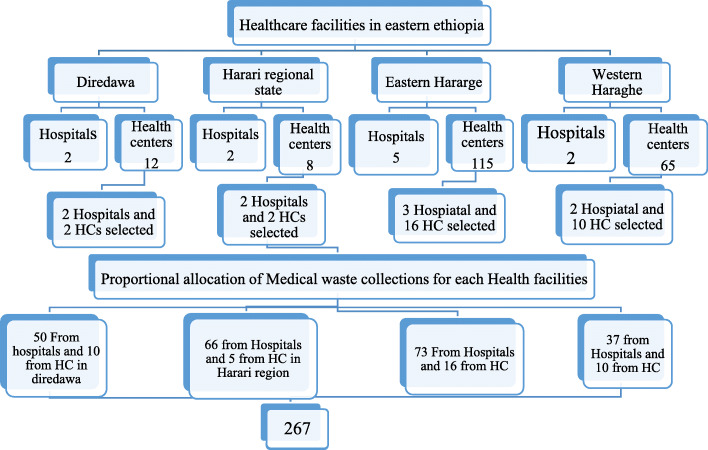
Flow diagram of Health facility and participant selection for HBV infection study in Eastern Ethiopia, 2018 (NB: HC; Health center)

A facility-based cross-sectional study was conducted in public health facilities of Harari region, Diredawa administration, and east and west Hararghe zones of Oromia regional state, eastern Ethiopia, from March to June 2018. According to the data obtained from the respective health offices, there were around 211 public health facilities (11 hospitals and 200 health centers) in the study area (115 health centers and 5 hospitals are found in east Hararghe, 65 health centers and 2 hospitals in west Hararghe, 12 health centers and 2 hospitals in Diredawa, and 8 health centers and 2 hospitals in Harar) (Fig. 1).

### Participants and sampling procedures

Multistage sampling technique was used and facilities were then stratified into hospitals and health centers. Eleven Hospitals and 30 health centers were selected from a total of 211 health facilities in eastern Ethiopia by lottery method. Sample size was determined using single population proportion formula for both objectives. Accordingly, sample size was calculated considering a beta of 80% and an alpha of 0.05% and design effect of 1.5. It was estimated 267 samples were needed which was obtained with the second objective (risk factors for HBV infection) from previous study by Anagaw et al. [12]. The Study participants were then proportionally allocated for respective selected health institutions based on the number of study population working at each selected health institution. Registered lists of study participants were used as sampling frame.

### Inclusion and exclusion criteria

All medical waste collectors who were working at health facilities and healthy enough to provide information were included. Those medical waste collectors who were critically ill, have mental problem and have not engaged/exposed to medical waste management were excluded.

The primary outcome of this study was prevalence of HBV infection and our secondary outcome were factors associated with HBV infections such as socio demographic characteristics, occupational exposure to medical waste, immunization with hepatitis B virus vaccines, blood transfusions, multiple sexual partners, and family history of liver disease and others non-occupational risk factors.

### Data collection and data quality control

A pretested structured questionnaire was used for data collection from medical waste collectors. Five Nurses and five Public health professionals were recruited to facilitate data collection. The questionnaire was developed after reviewing different literature and pretested on 5% of sample size working at hospital which were not included in the study [11, 12, 21, 22]. The questionnaire was translated to local language (Afan Oromo and Amharic). During data collection, the data was checked for its completeness each day by supervisors and investigators after it was collected. For blood sample collection, all material we used were tested before purchase. Blood sample was collected with standard precaution and centrifuged to separate serum from whole blood immediately after collection of blood.

### Blood sample

Five laboratory professionals were recruited to collect blood sample. 2.5 ml of venous blood samples was collected in tubes aseptically after written consents was obtained from study subjects. Serum was separated by centrifugation at 3000 r/min for 5 min after the blood was clotted. The blood samples were labeled with unique identification numbers. The serum was separated by centrifugation and was placed into Eppendorf tubes. The serological tests were performed using hepatitis B surface antigen rapid test kits by Wondfo one step HBsAg test strip according to the manufacturer’s manual (Guangzhou Wondfo Biotech Co., Ltd. (Wondfo)). This test strip has sensitivity and specificity of 96.2 and 99.3% respectively [23].

### Data processing and analysis

Collected data was entered into Epi-data Version 3.1 and exported to SPSS version 22 statistical packages for analysis. Descriptive summary measures were made using frequency table, percentage and mean and standard deviation. Chi-square and Fisher exact tests were used to assess risk of association.

The variables with a *p*-value of less than 0.2 in the bivariate analysis were included in the multivariable analysis. Multivariable logistic regression (adjusted odds ratio with 95% confidence interval) analysis was employed to identify independent variables associated with HBV infections adjusting for other variables. Model fitness was checked using Hosmer Lemeshow goodness of fit and variables with *P*-value < 0.05 at 95% CI was declared statistical significance.

#### Ethical considerations

The study protocol was approved by Institutional Health Research Ethics Review Committee (IHRERC) of Haramaya University, College of Health and Medical Sciences (CHMS) and permission for study proceeding was accepted by respective health institutions. Then, written and signed informed consent was obtained from each participant. In order to protect the confidentiality of the information, names and identity number of participants were not included in the questionnaires. Results were communicated to study subjects and subjects with positive test were refereed for further investigations and follow-ups.

## Result

### Socio-demographic characteristics of the study participants

A total of 260 (97.38%) medical waste collectors have participated in this study. Seven participants refused to participate in this study. Two hundred thirty-seven (91.2%) participants were females (Table 1). The median age of the study participant was 29 years (IQR = 8). The majority (58.1%) of the study participants were married whereas the median years of service was 4 years (IQR = 4) (Table 1).

**Table 1:** Sociodemographic characteristics the study participants in public health facilities in eastern Ethiopia, 2018

Variables	Category	Frequency	Percent (%)
Sex	Male	23	8.8
	Female	237	91.2
Educational status	Unable to read and write	21	8.1
	Can read and write	30	11.5
	Grade 1–8	72	27.7
	Grade 9–10	79	30.4
	Grade 12	26	10
	Diploma and above	32	12.3
Marital status	Single/unmarried	79	30.4
	Married	151	58.1
	Divorced	25	8.7
	Widowed	5	1.9
Religion	Orthodox	141	54.2
	Muslim	101	38.8
	Protestant	18	6.9
Ethnicity	Amhara	129	46.6
	Oromo	114	43.8
	Others^*^	17	6.6
Average monthly income	Earns between 20 and 55 US$	244	93.8
	Earns between 56 and 107 US$	16	6.2

NB: Average monthly income was calculated based on Ethiopian government employee monthly salary in Ethiopian birr and converted to United states dollar

Others* = Harari, Tigray, Gurage

US$: United states dollar

### Prevalence of hepatitis B infection among study subjects

According to this study, 20.4% [95% CI; 15.8, 25.6] of the respondents are found positive for HBsAg test. There was no statistically significant difference in the rate of HBV infection between socio-demographic characteristics of the study participants (*P* > 0.05). Of note, statistically significant difference was noted in exposure to blood ungloved (*P* = 0.002), history of multiple sexual partner (*P =* 0.001), and being unvaccinated for HBV (*P =* 0.001).

### Knowledge, training and waste handling practices

According to this study, 51.9% (135) of the study participants have poor knowledge on universal precaution guideline and only 39.6% (103) have participated in training program about infection prevention or universal precaution. More than two third (76.9%) said they cleaned their hand after touching or collection of blood or other body fluids. Two hundred two (85.4%) study subjects said they wear glove consistently for personal protective purpose. On the other hand, about one half, 43.1% of the study participants have history of exposure to any types of body fluids like waste contaminated by body fluids (blood, peritoneal, pericardial, pleural, synovial, CSF amniotic fluids and others). Out of these study participants, 61.61% have history of exposure to blood ungloved. The majority 52.69% (137) of the study participants said they have recapped needle that were in appropriately recapped. About 41.2% (107) of study participants said they had history of sharp material injury, and 38.5% (100) had history of needle stick injury. Fourth eight (44.86%) of the study participants had had sharp injury twice (Table 2). There was no significant difference in HBsAg positivity with respect to frequency of sharp injury (*p* value > 0.05).

**Table 2:** Frequency of sharp injury among medical waste handlers/collectors in public health facilities in eastern Ethiopia, 2018

Frequency of sharp injury		Frequency	Percent (%)
Valid	Once	41	38.31
	Twice	48	44.86
	Three times	7	6.54
	Several times	11	10.28
	Total	107	100

Thirty-five (13.5%) study subjects said they had history of surgical operation. Approximately 9% (23) of the study participants had participated in blood transfusion. Furthermore, 11.5% (30) of the study participants said they have been diagnosed with jaundice and/or liver diseases, and 12.3% (32), and 22.7% (59), had history of tattooing and tooth extraction respectively. From the total study subjects, 8.1% (21) said that they had history of multiple sexual partners in the last 1 year out of which 3.8% (10) had sex with commercial sex workers. Eleven study subjects (4.1%) said that they had family history of chronic liver disease. Among the total study subjects, 51.2% (133) said that they have been vaccinated for HBV.

### Factors associated with HBV infection among the study population

Sex, wearing personal protective equipment (PPE), exposure to blood ungloved, history of sharp injury, history of operation/surgery, history of blood transfusion, history of tattooing, history of multiple sexual partners, and vaccination status for HBV were entered into the model as these variables had *p*-value less than 0.2 in bivariate analysis.

Study subjects who have never been vaccinated for HBV were 6.35 (AOR = 6.35; 95%CI: [2.53–15.96]) times more likely to be positive for HBsAg compared to those who have been vaccinated for HBV (Table 3). Medical waste collectors who had history of blood transfusion (received) and tattooing were 3.54 and 2.86 times (AOR; 3.54; 95%CI, [1.02–12.24]; AOR = 2.86; 95% CI: [1.12–7.27]) more likely to be positive for HBsAg than their counterparts respectively (Table 3). Furthermore, the odds of being positive for HBV infection is 10.28 (AOR = 10.28; 95%CI: [4.16–25.38]) times higher among medical waste collectors who had multiple sexual partners in the last 1 years than those who had no history of multiple sexual partners.

**Table 3:** Factors associated with HBsAg positive status among medical waste handlers/collectors in Public health facilities in eastern Ethiopia, 2019 (*n* = 260)

Variables	Response	HBsAg status		COR [95%CI]	AOR [95%CI]	P-value
		Positive	Negative			
Sex	Male	8	15	1	1	
	Female	45	192	2.28 (0.91–5.70)	0.53 [0.16–1.69]	0.28
Wear PPE	Yes	45	190	1	1	
	No	8	17	1.99 (0.81–4.89)	1.34 [0.46–3.91]	0.59
Ever vaccinated for HBV	Yes	13	120	1	1	
	No	40	87	4.24 (2.14–8.41)	6.35 [2.53–15.96]	0.001
History of sharp injury	No	26	127	1	1	
	yes	27	80	1.65 (0.90–3.02)	1.54 [0.73–3.27]	0.25
History of operation/surgery	No	49	176	1	1	
	Yes	4	31	0.46 (0.16–1.38)	0.89 [0.24–3.26]	0.86
History of blood transfusion	No	45	192	1	1	
	Yes	8	15	2.28 (0.91–5.70)	3.54 [1.02–12.24]	0.046
History of tattooing	No	41	187	1	1	
	Yes	12	20	2.74 (1.24–6.04)	2.86 [1.12–7.27]	0.03
History of multiple sexual partner	No	32	191	1	1	
	Yes	21	16	7.83 (3.70–16.60)	10.28 [4.16–25.38]	0.001
Exposure to blood ungloved	No	30	161	1	1	
	Yes	23	46	2.68 (1.42–5.06)	1.69 [0.77–3.71]	0.92

## Discussion

This study demonstrated that the seroprevalence of hepatitis B virus infection was high (20.4%). This finding is much higher than a study from Tripoli Libya [21], northern Ethiopia [12], southern Ethiopia [1], North-west Ethiopia [24] and Addis Ababa [11]. Low training coverage, being unvaccinated, history of multiple sexual partner and poor knowledge on universal precaution guideline among our study groups may explain this high infection rate.

This study revealed that the odds of being positive for HBsAg was higher among medical waste collectors who have never been vaccinated compared to those who have been vaccinated for HBV. This finding is similar with study conducted in Thailand [25], Libya [21] and northern Tanzania [26]. HBV vaccines was found to be protective against HBV infection [26] which constituted a land mark of great importance for workers in health care facilities including medical waste collectors. However, lack of universal availability of the vaccine for adult population in Ethiopia [12, 27] may explain this finding. In addition, this study finding indicates the health facilities in this study do not take appropriate steps to ensure workers safety necessary to avoid occupational hazards. This indicates the need for training on universal precaution guideline, health promotion and scale up of HBV vaccination.

Blood transfusion (receiving) has been found to be a risk factor for HBV infection among our study group. Those who received blood donation were 3.5 more likely to be positive for HBV than their counterpart. This finding is similar with study in Spain [28], Japan [29], Nigeria [30, 31], Conakry Guinea [32]. Despite increasing screening of blood for HBV before giving to patients, the remaining residual risk of transfusion transmission of HBV infection remain high due to suboptimal screening practice [31, 33]. This finding is in contrast to the current practice by most of the local blood bank centers in Ethiopia which screen blood for safe blood transfusion to prevent transfusion-transmitted infections [34]. The national policy of Ethiopia on communicable disease prevention and control also give special emphasis for screening of donors’ blood prior to blood transfusion [35, 36]. In addition, this finding is in contrast to study by Alter where transmission of HBV via transfusion or transplant has been virtually eliminated in countries that test donors for HBsAg and virally inactivate plasma-derived products [6].

Literature indicated that HBV transmissions can result from non-compliance with aseptic techniques during procedures such as surgery or tattooing that resulted in cross-contamination of medical equipment and devices [37, 38]. Previous history of tattooing significantly increased the odds of HBV infection by 3 times than those with no history of tattooing. Previous studies have also identified that the rate of lifetime exposure to HBV infection was significantly higher among medical waste handlers who had received a tattoo [1].

Perinatal and sexual exposures to HBV are also highly efficient modes of transmission [6, 39–41]. In this study, those who had multiple sexual partners in the last 1 year were almost 10 times more like to be infected by HBV than their counterparts. This finding is similar to studies conducted in Tigray Ethiopia [28], North-west Ethiopia [24, 42] Slovakia [43] and in Iran [44]. This is a scientifically well-established fact where parental and sexual route play an important role in the transmission of HBV.

Though not significant, training provided by health facilities in this study area to medical waste collectors were inadequate and lack regularity. This is similar with other study finding in Ethiopia [12]. Sufficient training should be given for medical waste collectors on waste handling, internal transport, spill management and storage requirements for the different types of wastes arising at the facility to minimize the risk of injury associated with waste handling.

None of socio-demographic characteristics and occupational exposures such as not wearing personal protective equipment, exposure to blood ungloved, history of sharp injury, surgical operations, liver disease, tooth extraction, blood transfusion, and family history of liver disease were shown statistically significant association with HBV infection in this study. However; the rate of occurrence of body fluid exposure to mucous membranes and blunt-penetrating injuries in work sites were higher. Furthermore, training on universal precaution guideline was low where on 39.6% were trained for short duration.

### Strength and limitation of the study

This study is a large-scale study including many health facilities and geographic areas which may increase its representativeness of the finding. However, we recognized certain limitations including insufficient immunoassay logistics supply which restricted this study to provide data on other HBV serological markers as HBeAg, IgM and IgG anti-HBc”. In addition, the use of rapid tests in the absence of confirmatory conventional immunoassays may be also a limitation. This limited the study classifying whether infections are acute or chronic and whether the degree of infectivity correlates with a high level of HBV replication or not.

## Conclusions

The prevalence of hepatitis B virus infection among medical waste collectors was higher in public health facilities in eastern Ethiopia compared to other findings. Occupational exposure due to being unvaccinated, infection due to tattooing, blood transfusion and history of multiple sexual partners were significant factors associated with high HBV infection. Medical waste collectors also had poor awareness of viral hepatitis B infection prevention and had low training on universal precaution guidelines. These findings highlight the need for continues improvement of working environment of medical waste collectors on provision of occupation precautions and health promotion. These health institutions should promptly adopt procedures and policies for occupation protection of their workers through continuous training and health promotion to improve their health knowledge. Effective scale up of free hepatitis B vaccine and adoption of more safe ways for waste collection could be considered in order to control the risk of HBV infection.

## Supplementary Information

**Additional file 1.**

## Data Availability

Most of the data generated or analyzed during this study are included in this published article. The full datasets used and/or analyzed during the current study is available from the corresponding author on reasonable request.
